# Targeted lipidomics between hPheo1 and SDHB KD cells reveal changes in bioactive lipids and PKC with polyamine pathway inhibition

**DOI:** 10.14814/phy2.70894

**Published:** 2026-06-05

**Authors:** Abdel A. Alli, Niharika Bala, Yiling Xu, Nancy D. Denslow, Hans K. Ghayee

**Affiliations:** ^1^ Department of Medicine, Division of Nephrology, Hypertension, and Renal Transplantation, College of Medicine University of Florida Gainesville Florida USA; ^2^ Department of Physiology and Aging, College of Medicine University of Florida Gainesville Florida USA; ^3^ Department of Medicine, Division of Endocrinology University of Florida and Malcom Randall VA Medical Center Gainesville Florida USA; ^4^ Department of Physiological Sciences and Center for Environmental and Human Toxicology University of Florida Gainesville Florida USA

**Keywords:** diethylnorspermine, paraganglioma, pheochromocytoma, phospholipids, protein kinase C, succinate dehydrogenase B subunit

## Abstract

Pheochromocytoma and paraganglioma with a succinate dehydrogenase B subunit (SDHB) pathogenic variant are associated with a significant chance for metastasis. Polyamine pathway inhibitor N1,N11‐diethylnorspermine (DENSPM) was previously shown to inhibit cell growth in progenitor cells derived from a human pheochromocytoma (hPheo1). Here, we hypothesized cell death associated with DENSPM treatment due to altered lipid metabolism affects protein kinase C (PKC). From targeted lipidomics analysis, baseline bioactive lipids that are distinct between the hPheo1 WT and SDHB KD cells are PE(P‐18:1/16:0), PI(18:1/20:3), LPE(18:0), and LPE(22:4). With DENSPM treatment, the concentrations of multiple plasmanyl phosphatidylethanolamines (PE‐O), sphingomyelins (SM), and hexosylceramides (HCER) increased, while the concentrations of several plasmenyl phosphatidylethanolamines (PE‐P) were decreased in hPheo1 WT and SDHB KD cells. The differences in PE‐Ps, PE‐Os, and SMs after DENSPM treatment compared to the vehicle treatment were greater in the SDHB KD cells compared to the hPheo1 WT cells. Basal PKC alpha protein expression was increased in SDHB KD cells compared to hPheo1 WT cells. The protein expression of both PKC alpha and delta was significantly decreased with DENSPM treatment in both cell lines. DENSPM changed pro‐caspase‐3 and cleaved caspase‐3. These data suggest ether phospholipids are biomarkers of DENSPM mediated cell apoptosis through a PKC dependent mechanism.

## 1 INTRODUCTION

Pheochromocytomas (PCCs) and paragangliomas (PGLs) (PCPG's) are rare neuroendocrine tumors (Juhlin, 2021). However, PCPG patients with mutations in the succinate dehydrogenase subunit B (*SDHB*) gene have a greater incidence of metastatic disease. Moreover, metastatic PCPG's usually present as advanced tumors with limited treatment options. Polyamines are polycations that play an important role in the regulation of ion channels (Dhara et al., 2020; Forsythe, 1995; Williams, 1997) receptors (Bowie, 2018; Rock & Macdonald, 1995), protein synthesis (Igarashi et al., 1988), cell proliferation, and differentiation (Heby, 1981). Elevated cellular polyamine levels have been reported in the tumorigenesis of various cell types (Jiang, Choi, Khan, et al., 2007). The polyamine pathway has recently been investigated as a therapeutic strategy to suppress cancer cell growth (Evageliou et al., 2016). Several studies have investigated the inhibition of cellular growth (Akinyele & Wallace, 2021; Huang et al., 2003; Ray et al., 2001) and changes in intracellular signaling cascades (Facchini et al., 2005; Keledjian et al., 2012; Liu et al., 2003) of different cell types after inhibiting the polyamine pathway.

In PCPG, it has been shown that the polyamine analogue N^1^,N^11^‐diethylnorspermine (DENSPM) inhibits cell growth in hPheo1 cells and suppresses growth in Xenograft mice (Rai et al., 2020). Earlier studies indicate the response of DENSPM in tumors is associated with the induction of the polyamine catabolic enzyme, spermine/spermidine N1‐acetyltransferase (SSAT) leading to the conversion of spermine and spermidine to their acetylated forms (Gabrielson et al., 2004). Acetylated polyamines have been shown to be involved in the generation of H_2_O_2_ resulting in cellular oxidative stress and cell death (Ha et al., 1997; Pledgie et al., 2005). In glioblastoma cells, DENSPM treatment results in microtubule collapse and abnormal mTOR protein expression (Jiang, Choi, Hu, et al., 2007). Studies on melanoma cells showed DENSPM may activate the mitogen‐activated protein kinase (MAPK) pathway (Chen et al., 2003). In addition to regulating signaling pathways in cancer cells, DENSPM may regulate membrane lipids that are important in cancer cell proliferation.

The analysis of lipid profiles in PCPG patients (Berent et al., 1987) and cultured PCC cells has been studied (Yun et al., 2006). Yun et al. showed the bioactive lipid lysophosphatidylcholine (LPC) stimulates phospholipase D2 (PLD2) activity in rat PC12 cells. De novo synthesis of diacylglycerol (DAG) and hydrolysis of phosphatidylinositol (PI) are stimulated by nerve growth factor in rat PCC cells (Li & Wurtman, 1998). In addition to LPC and DAGs, there are several other bioactive lipids that play important roles in PCC cells. Phosphatidylethanolamine (PE) tends to exist in diacyl, alkylacyl, and alkenyl forms, with the last two forms being ether lipids, Plasmanyl (PE‐O)/Plasmenyl (PE‐P) phosphatidylethanolamines, respectively. PE can also lose one of its fatty acids to become lyso‐phosphatidylethanolamine (LPE). PE‐O, PE‐P, and LPE are among the bioactive forms of phosphatidylethanolamines found in cell membranes (Dawaliby et al., 2016; Dean & Lodhi, 2018; Lee, 1998). LPE was shown to induce chemotactic migration and cell invasion in SK‐OV3 cells (Park et al., 2007). The ability of DENSPM to alter the concentrations of these lipids in the plasma membranes of hPheo1 wild‐type (WT) and *SDHB* knockdown (*SDHB KD*) cells or the role of these bioactive lipids in the growth of these cells has not been investigated.

Multiple studies have demonstrated bioactive lipids regulate protein kinase C (PKC) in cancer cells (Cooke et al., 2019; Garcia‐Bermejo et al., 2002). Additionally, PKC has been shown to play multiple roles in PCC cells (Gardner & Olah, 2003; Han et al., 2002). PKC activation was shown to enhance production of the second messenger cyclic AMP (cAMP) in rat PCC cells (Hollingsworth et al., 1986). PKC‐dependent phosphorylation was reported to regulate the enzyme that catalyzes phosphatidylcholine hydrolysis, phospholipase D (PLD), in PC12 cells (Han et al., 2002). The regulation of PKC by DENSPM treatment has not been investigated in human cells derived from PCC.

To fill these knowledge gaps outlined here, in this study, we tested the hypothesis that the mechanism of action of DENSPM involves changes in the concentration of bioactive lipids within the membranes, PKC expression, and apoptosis markers of hPheo1 WT and *SDHB KD* cells. The overall goal of this study was to investigate the potential utility of DENSPM as an anticancer drug.

## 2 MATERIALS AND METHODS

### 2.1 Reagents

DENSPM was provided as a kind gift from Professor Raymond J Bergeron, Eminent Scholar, Department: Medicinal Chemistry.

### 2.2 Cell culture

hPheo1 WT cells (female) and hPheo1 *SDHB KD* cells (female) were cultured in RPMI 1640 media supplemented with 10% fetal bovine serum (FBS) after evaluating multiple lots of serum for optimal cell viability. The media were exchanged every 3 days and the cells were maintained in a 5% CO_2_ humidified incubator. All cell culture experiments were performed using cells from at least 3 different passages of each cell type.

### 2.3 MTT assay

An MTT assay (4890‐025‐k; R&D Systems; Minneapolis, MN) was performed to measure cell proliferation. Briefly, hPheo1 WT and hPheo1 *SDHB KD* cells were plated in 96‐well flat‐bottom tissue culture plates at densities ranging from 1,250 to 25,000 cells per well in 100 μL of complete culture medium and incubated for 24 h to allow adherence and recovery. Subsequently, 10 μL of MTT reagent was added to each well, and plates were incubated for 12 h at 37°C. After visible formation of purple crystals, 100 μL of detergent reagent was added, and the plates were kept in the dark at room temperature for 2 h. Absorbance was measured at 570 nm using a microplate reader. Blank wells containing only medium were used as controls, and mean absorbance values (after blank subtraction) were plotted against cell number to identify a linear response range (target A570 = 0.75–1.25). Three technical replicates were used and three independent experiments were performed.

### 2.4 BCA protein assay

A BCA protein assay (Cat. No. A55860) (ThermoFisher Scientific, Waltham MA) was performed after preparing 9 standards from a stock concentration of 2 mg/mL of BSA. The samples were diluted 1:10 in reagent grade water before adding a mixture of BCA reagent A and B. The plate was read at 570 nm.

### 2.5 Lipid extraction and lipidomic analysis

Lipids were extracted and analyzed as previously described by our group (Alli et al., 2023; Lopez et al., 2022) with the following modifications. Briefly, hPheo1 WT cells and *SDHB KD* cells were treated with 10 μM DENSPM for 72 h. Cell lysates were collected after scraping the cells in EasyPep buffer (ThermoFisher Scientific). The cells lysates were subject to ultracentrifugation for 30 min at 106,300×*g* using a T70.1 rotor (Beckman Coulter; Brea, CA). The resulting particulate/membrane fraction was reconstituted in EasyPep buffer. Next, membrane fractions containing equivalent amounts of protein (32.5 ug) were extracted for lipids following the method of Bligh and Dyer (Bligh & Dyer, 1959). Samples (*n* = 3 biological replicates from each group) were adjusted to 1 mL with H_2_O, and 2.9 mL of a mixture of methanol: methylene chloride (2:0.9 v/v) was added to the glass extraction tubes followed by vortexing for 30 s. Next, 50 μL of 5X diluted EquiSPLASH Lipidomix internal standards (Avanti, Alabaster AL) were added to each sample. The mixture was again vortexed and incubated for 30 min at room temperature. Then, 1 mL H_2_O and 0.9 mL of methylene chloride were added to each sample. The samples were gently inverted 10 times and then centrifuged at 200*×g* for 10 min. The lower phase (methylene chloride) was collected, concentrated to dryness under a N_2_ stream and reconstituted into 50 μL methanol. The lipid extracts were analyzed by ultra‐high performance liquid chromatography (UHPLC, Shimadzu Co, Kyoto, Japan) linked to a QTRAP tandem mass spectrometer (QTRAP 6500, ABSCIEX, Redwood Shores, CA, USA). Chromatography was performed on a Luna NH2 column (3 um, 2.1 X 100 mm) (Phenomenex, Torrance, CA, USA) using a binary gradient with mobile Phase A: Acetonitrile: Water (95:5) with 1 mM Ammonium acetate, pH  8.2, and mobile Phase B: Acetonitrile: Water (50:50) with 1 mM Ammonium acetate, pH = 8.2. The mass spectrometer was operated with a scheduled multiple reaction monitoring (MRM) method in both positive and negative modes. The following settings for the mass spectrometer were used: Declustering potential 60 or −80; Entrance potential 10 or −10; Collision energy between 25 and 43 in positive mode and −40 and −61 in negative mode; Collision cell exit potential 15 or −15; Ion spray voltage was 5500 kV or −4.5 kV; curtain gas was 35 psi; GS1 was 50 psi; GS2 was 60 psi; and the source temperature was 550°C. The operating software was Analyst (SCIEX, v1.6.2), and MultiQuant (SCIEX, v3.0.3) was used for peak inspection and quantification. Lipid values missing in more than half the samples were replaced with 1/5 the lowest value for each lipid.

### 2.6 SDS PAGE and Western blotting

Fifty micrograms of protein from membrane fractions of hPheo1 WT and *SDHB KD* cell lysate were resolved on 4%–20% gradient gels at 200 V for 1 h. The proteins were transferred onto nitrocellulose membranes in chilled Towbin buffer (25 mM Tris, 192 mM glycine, 20% methanol (vol/vol)). The blots were stained with Ponceau solution and then blocked in a solution of 5% nonfat dry milk in 1XTBS for 1 h. After a series of washes with 1XTBS the blots were incubated in primary antibody (anti‐cleaved caspase 3 (9661; Cell Signaling Tech) (Danvers, MA), anti‐pro‐caspase 3 (66,470; Proteintech), anti‐PKC alpha (59,754; Cell Signaling Tech), or anti‐PKC delta (9616; Cell Signaling Tech) diluted 1:1000 in 5% BSA 1XTBS solution and incubated at 4°C while rocking overnight. The blots were subject to a series of three washes in 1XTBS before being incubated in goat anti‐rabbit secondary antibody (Cat. No. 1706515) (BioRad) diluted 1:3000 (prepared in blocking solution) at room temperature while rocking for 1 h. Blots were then incubated in ECL solution (Cat. No. 1705061) (BioRad) before being developed on a BioRad imager.

### 2.7 Immunofluorescence microscopy

Cells were cultured on 35 mm glass‐bottom dishes (Mattek; Ashland, MA, USA). After overnight treatment, the cells were washed with 1XPBS and then fixed for 10 min with ice‐cold methanol/acetone (1:1 v/v). After two washes with 1X PBS, the cells were incubated in 2.5% normal horse serum for 20 min at room temperature. Next, they were incubated with primary antibodies prepared in ultrapure 1X PBS at a 1:200 dilution for 45 min. The cells were then washed twice with 1X PBS and incubated with secondary antibodies (Vector Fluor Duet reagent, Vector Laboratories, USA). Finally, after two additional washes with 1X PBS, the cells were cover‐slipped with antifade mounting media (Thermofisher, Waltham, MA) containing DAPI and imaged using an Olympus microscope.

### 2.8 Statistical analysis

Data are presented as Mean ± SEM and differences with a *p*‐value of less than 0.05 were considered statistically significant. Comparisons were made between the groups using a one‐way ANOVA in SigmaPlot 15.0 software or MetaboAnalyst 6.0 software (Pang et al., 2024).

## 3 RESULTS

### 3.1 Assessment of cell proliferation in hPheo1 cells and SDHB KD cells

hPheo1 cells and *SDHB KD* cells were plated at different densities and cell proliferation was measured by an MTT assay. *SDHB KD* cells showed a higher proliferation rate compared to hPheo1 cells at different densities (Figure S1).

### 3.2 Differences in lipid abundance in hPheo1 WT and SDHB KD cell membranes at baseline

**FIGURE 1:**
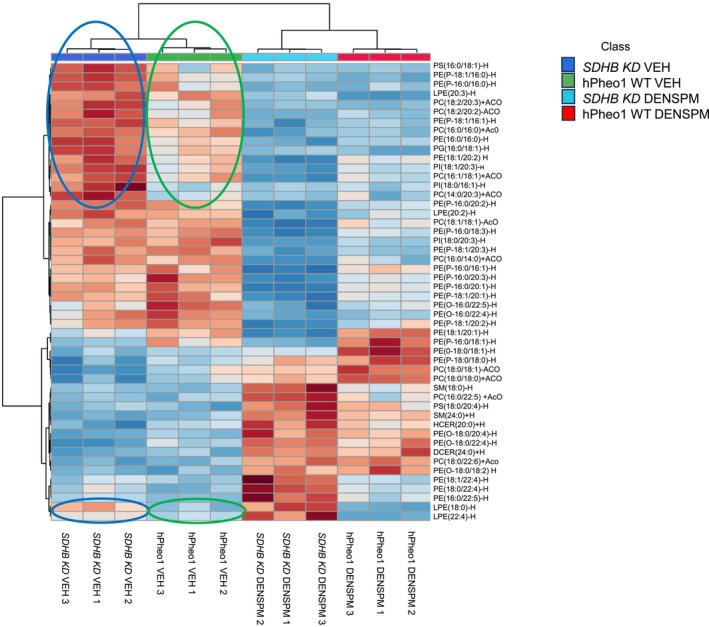
Heatmap showing the top 50 lipids that show differences in concentrations between hPheo1 WT and *SDHB KD* cells and between the two cell types treated with vehicle or DENSPM. Each colored cell on the map corresponds to a concentration value in the dataset. The raw lipidomics data were normalized to median with Pareto scaling. Data from *n* = 3 biological replicates (3 independent lipid sample preparations) from each of the 4 groups. A One‐way Analysis of Variance was performed with an adjusted *p*‐value cutoff of 0.001, followed by a Tukey's post‐hoc analysis.

Since SDHB inactivity can lead to metabolic changes, it is expected that lipids within the plasma membrane would be different in hPheo1 WT and *SDHB KD* cells. As shown in Figure 1, the heatmap of our lipidomics dataset showed differences (blue and green ovals) in the abundance of various lipids within membrane fractions of hPheo1 WT and *SDHB KD* cells. These lipids include PS(16:0/18:1), PE(P‐18:1/16:0), PE(16:0/16:0), PG(16:0/18:1), PE(18:1/20:2), PI(18:1/20:3), PC(14:0/20:3), LPE(18:0), and LPE(22:4) (Figure 1). The bioactive lipids that are distinct between the hPheo1 WT and *SDHB KD* cells are PE(P‐18:1/16:0), PI(18:1/20:3), LPE(18:0), and LPE(22:4).

### 3.3 Changes in lipid abundance in hPheo1 WT and SDHB KD cell membranes after DENSPM treatment

Our semi‐quantitative lipidomic analysis showed various lipid species from different lipid classes that were either increased or decreased in concentration in the membranes of hPheo1 WT and *SDHB KD* cells treated with DENSPM compared to vehicle. The heatmap in Figure 1, shows the top 50 lipids that are lower or greater in concentrations between the DENSPM and vehicle treatment groups and between the hPheo1 WT and *SDHB KD* cells.

### 3.4 DENSPM treatment reduces multiple PE‐P species while increasing multiple PE‐O species in hPheo1 WT and SDHB KD cells

**FIGURE 2:**
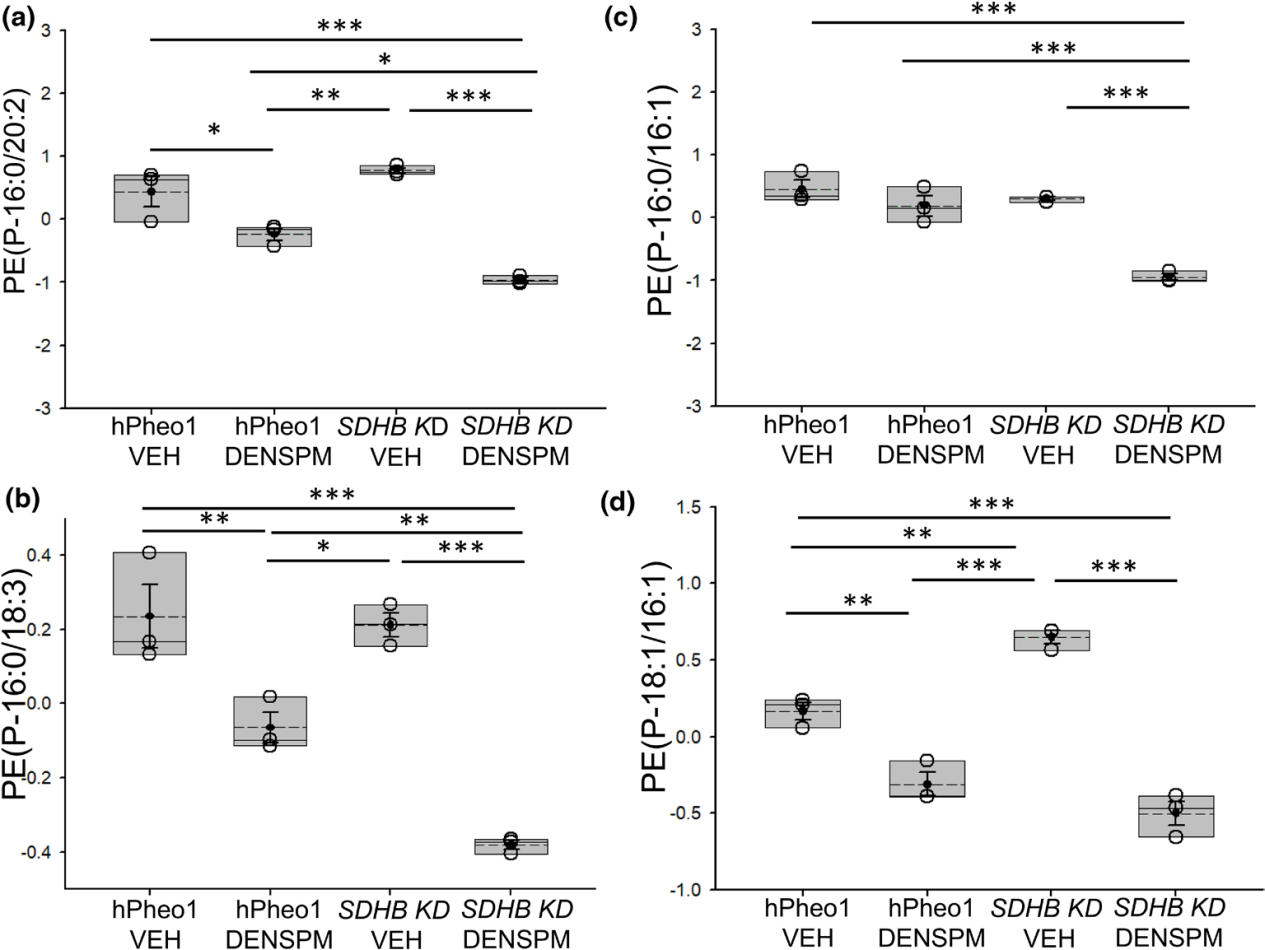
Concentrations of plasmenyl phosphatidylethanolamines (PE‐P) in hPheo1 WT and *SDHB KD* cells treated with vehicle or DENSPM. (a) PE(P‐16:0/20:2), (b) PE(P‐16:0/18:3), (c) PE(P‐16:0/16:1), (d) PE(P‐18:1/16:1). VEH, vehicle. DENSPM, N^1^,N^11^‐diethylnorspermine. Units for all lipids is μM. Data from *n* = 3 biological replicates (3 independent lipid sample preparations) from each of the 4 groups. * represents a *p*‐value <0.05, ** represents a *p*‐value <0.01, *** represents a *p*‐value <0.001. The solid line in the box plot represents the median while the dotted line represents the mean. The box represents the interquartile range from the 25th to the 75th percentile of the data, while the error bars inside the box represent the mean ± SEM.

Our targeted lipidomic study showed the basal concentration of several classes of lipids including different PEs are different between hPheo1 WT cells and *SDHB KD* cells (Figure 1). In addition, our lipidomics dataset showed multiple plasmenyl phosphatidylethanolamines (PE‐Ps) including PE(P‐16:0/20:2), PE(P‐16:0/18:3), PE(P‐16:0/16:1), PE(P‐18:1/16:1) were decreased in concentration in the membranes of hPheo1 WT as well as in the *SDHB KD* cells treated with DENSPM compared to vehicle treatment (Figure 2). Moreover, the decrease in concentration of the different PE‐Ps for the DENSPM treatment compared to the VEH treatment was greater in the *SDHB KD* cells compared to the hPheo1 WT cells (Figure 2).

**FIGURE 3:**
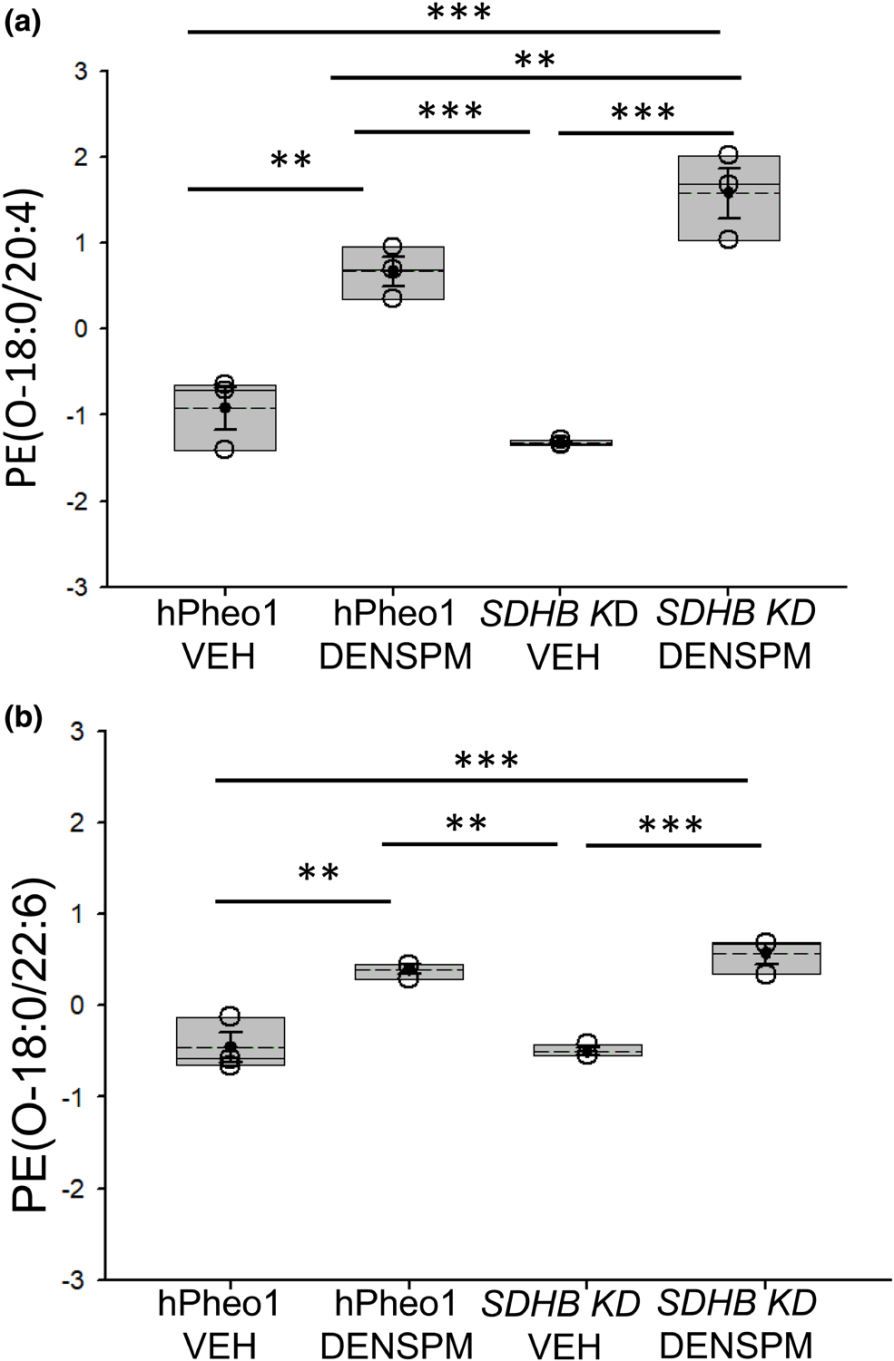
Concentrations of plasmanyl phosphatidylethanolamine (PE‐O) in hPheo1 WT and *SDHB KD* cells treated with vehicle or DENSPM. (a) PE(O‐18:0/20:4), (b) PE(O‐18:0/22:6). VEH, vehicle. DENSPM, N^1^,N^11^‐diethylnorspermine. Units for all lipids is μM. Data from *n* = 3 biological replicates (3 independent lipid sample preparations) from each of the 4 groups. ** represents a *p*‐value <0.01, *** represents a *p*‐value <0.001. The solid line in the box plot represents the median while the dotted line represents the mean. The box represents the interquartile range from the 25th to the 75th percentile of the data, while the error bars inside the box represent the mean ± SEM.

Conversely, multiple plasmanyl phosphatidylethanolamines (PE‐O), including PE(O‐18:0/20:4), and PE(O‐18:0/22:6) were increased in the membranes of hPheo1 WT and the *SDHB KD* cells treated with DENSPM compared to vehicle (Figure 3). In addition, the increase in concentration of the different PE‐Os for the DENSPM treatment compared to the VEH treatment was greater in the *SDHB KD* cells compared to the hPheo1 WT cells (Figure 3).

### 3.5 DENSPM treatment augments the concentration of multiple sphingomyelins in hPheo1 WT and SDHB KD cells

**FIGURE 4:**
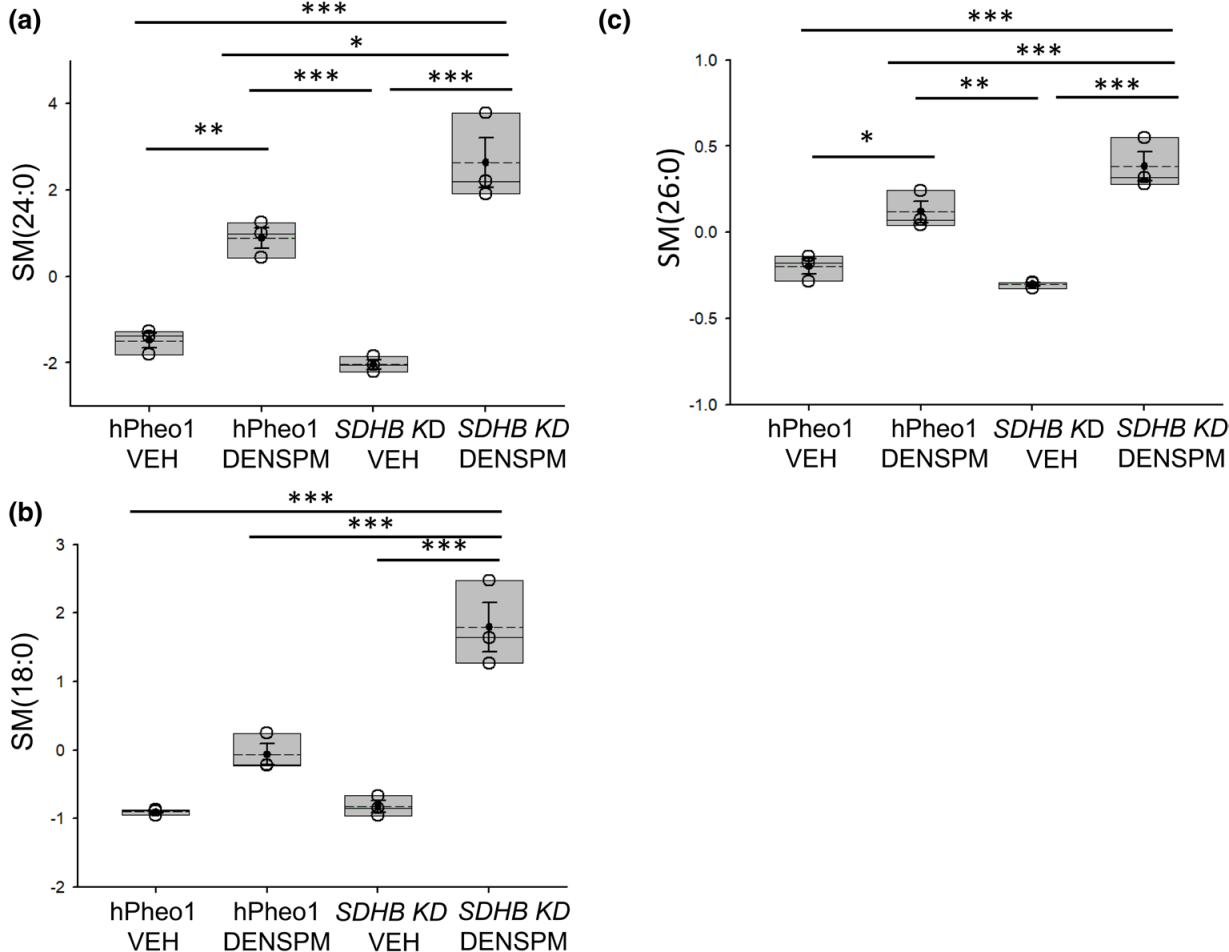
Concentrations of sphingomyelins (SMs) in hPheo1 WT and *SDHB KD* cells treated with vehicle or DENSPM. (a) SM(24:0), (b) SM(18:0), (c) SM(26:0). VEH, vehicle. DENSPM, N^1^,N^11^‐diethylnorspermine. Units for all lipids is μM. Data from *n* = 3 biological replicates (3 independent lipid sample preparations) from each of the 4 groups. * represents a *p*‐value < 0.05, ** represents a *p*‐value < 0.01, *** represents a *p*‐value < 0.001. The solid line in the box plot represents the median while the dotted line represents the mean. The box represents the interquartile range from the 25th to the 75th percentile of the data, while the error bars inside the box represent the mean ± SEM.

In addition to affecting the concentration of various PEs, our lipidomic study showed DENSPM augments the concentration of multiple sphingomyelins including SM(24:0), SM(18:0), and SM(26:0) in the membranes of both hPheo1 WT and *SDHB KD* cells (Figure 4). The increases in concentration of the different sphingomyelins for the DENSPM treatment compared to the VEH treatment were greater in the *SDHB KD* cells compared to the hPheo1 WT cells (Figure 4).

### 3.6 DENSPM treatment augments the concentration of multiple hexosylceramides in hPheo1 WT and SDHB KD cells

**FIGURE 5:**
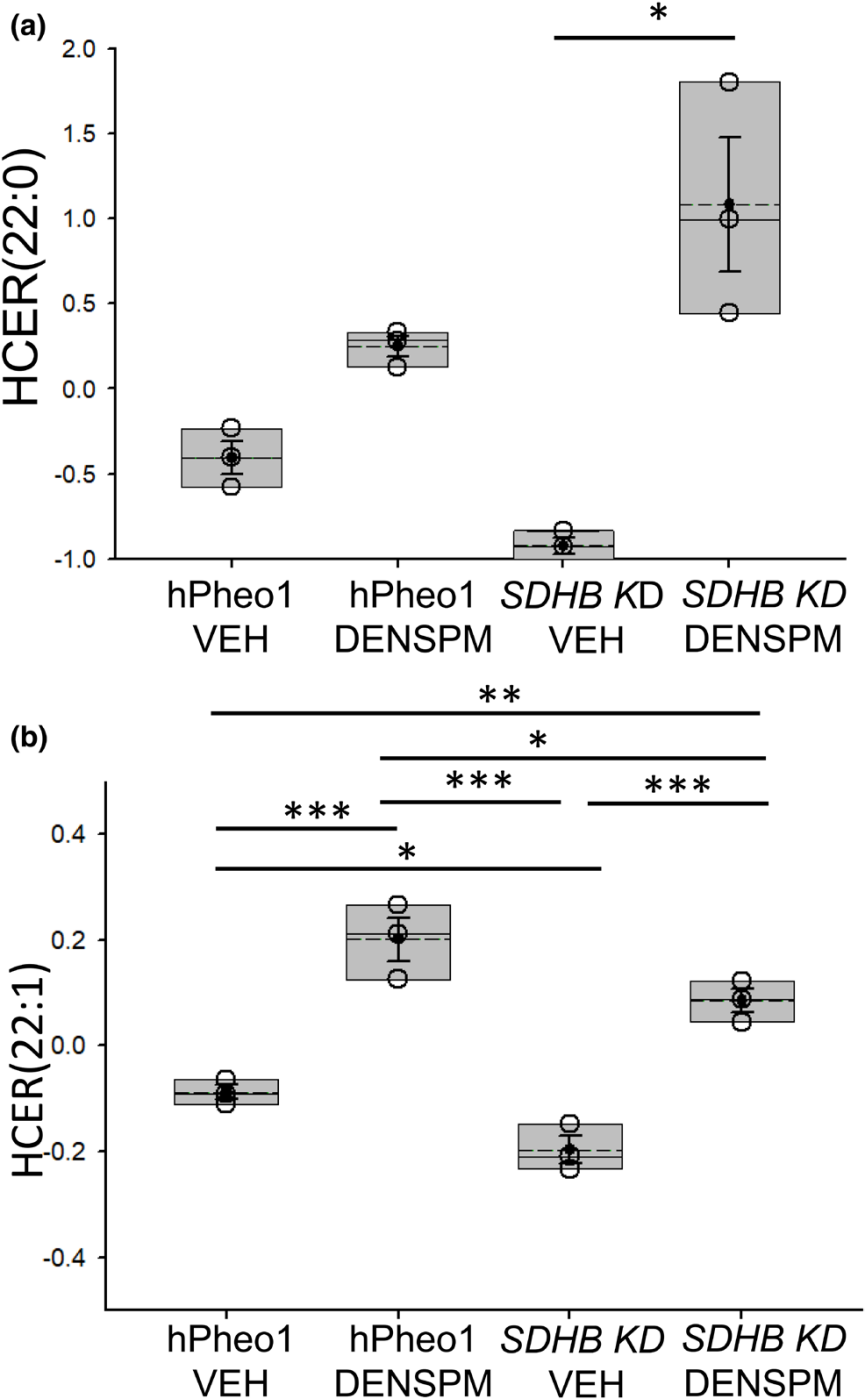
Concentrations of hexosylceramides (HCERs) in hPheo1 WT and *SDHB KD* cells treated with vehicle or DENSPM. (a) HCER(22:0), (b) HCER(22:1). VEH, vehicle. DENSPM, N^1^,N^11^‐diethylnorspermine. Units for all lipids is μM. Data from *n* = 3 biological replicates (3 independent lipid sample preparations) from each of the 4 groups. * represents a *p*‐value < 0.05, ** represents a *p*‐value < 0.01, *** represents a *p*‐value < 0.001. The solid line in the box plot represents the median while the dotted line represents the mean. The box represents the interquartile range from the 25th to the 75th percentile of the data, while the error bars inside the box represent the mean ± SEM.

Our lipidomic study also showed multiple hexosylceramides (HCERs), including HCER(22:0) and HCER(22:1), were augmented in the membranes of hPheo1 WT and *SDHB KD* cells treated with vehicle or DENSPM (Figure 5). For HCER(22:0), the increased concentration was greater for the *SDHB KD* cells compared to the hPheo1 WT cells (Figure 5).

### 3.7 DENSPM treatment decreases pro‐caspase‐3 and increases cleaved caspase‐3 in hPheo1 WT and SDHB KD

**FIGURE 6:**
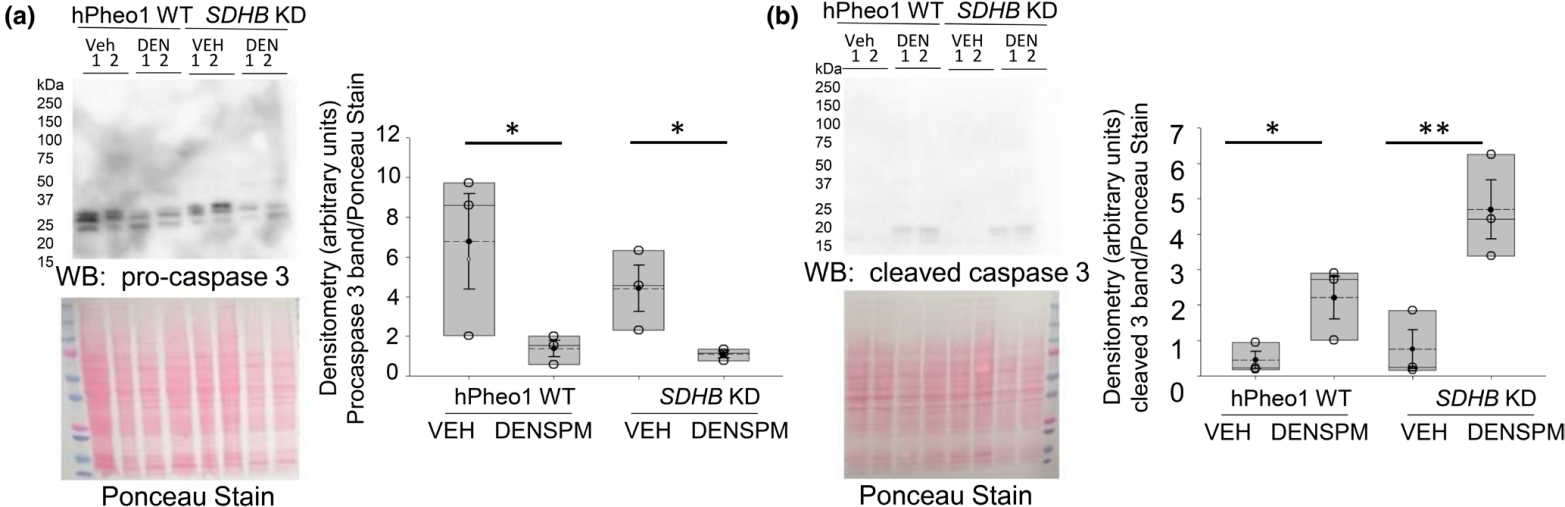
Representative Western blot analysis of pro‐caspase‐3 and cleaved caspase‐3 in hPheo1 WT and *SDHB KD* cells treated with vehicle or DENSPM. (a) Western blot (top) of pro‐caspase‐3 protein expression in hPheo1 WT and *SDHB KD* cells treated with vehicle or DENSPM. Ponceau Stain (bottom) used to assess lane loading. Densitometric analysis of the pro‐caspase 3 band normalized to the Ponceau stain, (b) Western blot (top) of cleaved caspase 3 protein expression in hPheo1 WT and *SDHB KD* cells treated with vehicle or DENSPM. Ponceau stain (bottom) used to assess lane loading.  Densitometric analysis of the cleaved caspase‐3 band normalized to the Ponceau stain. Data from 3 independent experiments. * represents a *p*‐value of <0.05. ** represents a *p*‐value of <0.01. The solid line in the box plot represents the median while the dotted line represents the mean. The box represents the interquartile range from the 25th to the 75th percentile of the data, while the error bars inside the box represents the mean ± SEM.

The changes in expression of the pro‐ and cleaved forms of caspase‐3 are considered established markers for dying or dead cells due to apoptosis. Here we show, compared to vehicle treatment, DENSPM treatment in *SDHB KD* cells, and also in hPheo1 WT cells causes a significant reduction in pro‐caspase‐3 (Figure 6a). Meanwhile, there was an increase in the expression of cleaved caspase‐3 in the *SDHB KD* cells treated with DENSPM compared to vehicle and the same trend was found in hPheo1 WT cells (Figure 6b).

### 3.8 DENSPM treatment reduces PKC alpha and delta protein expression

**FIGURE 7:**
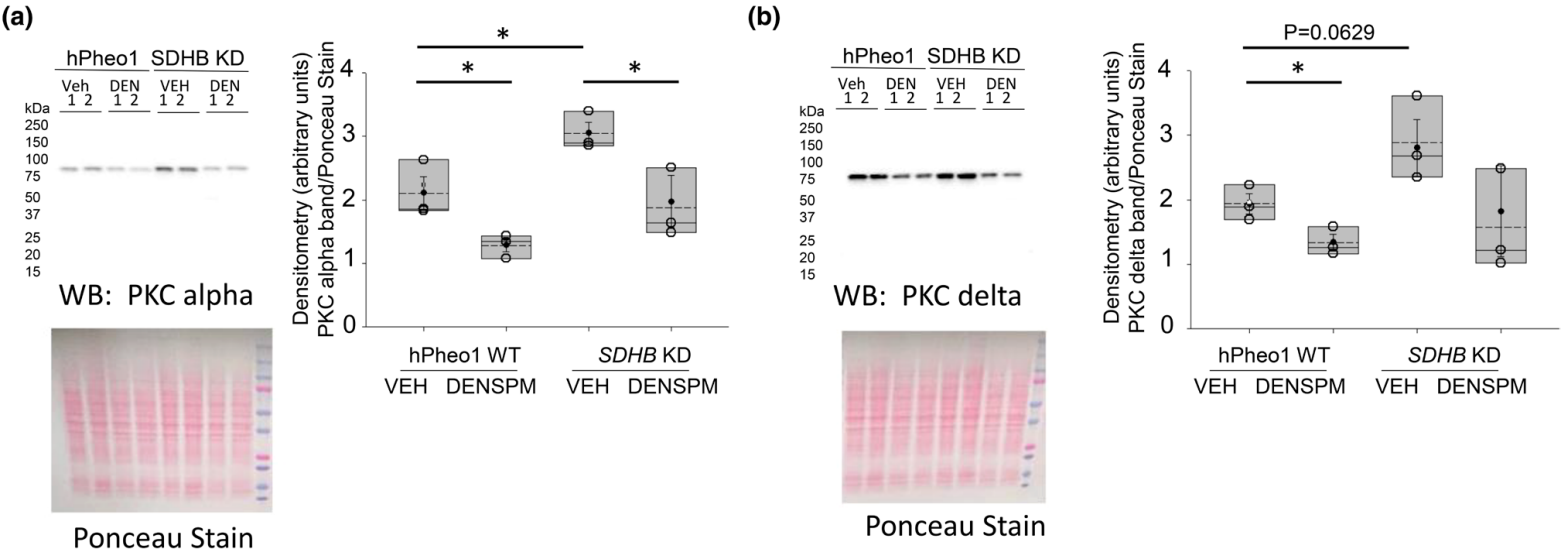
Representative Western blots and densitometric analysis of PKC isoform protein expression in hPheo1 WT and *SDHB KD* cells treated with DENSPM or vehicle. (a) Representative Western blot (top) of PKC alpha protein expression in hPheo1 WT and *SDHB* KD cells treated with vehicle (VEH) or DENSPM. Ponceau stain (bottom) used to assess lane loading. Densitometric analysis (right) of the PKC alpha blot. (b) Representative PKC delta Western blot (top) of PKC delta protein expression in hPheo1 WT and *SDHB* KD cells treated with vehicle (VEH) or DENSPM. Ponceau stain (bottom) used to assess lane loading. Densitometric analysis (right) of the PKC delta blot. Data from 3 independent experiments. * represents a *p*‐value <0.05. The solid line in the box plot represents the median while the dotted line represents the mean. The box represents the interquartile range from the 25th to the 75th percentile of the data, while the error bars inside the box represents the mean ± SEM.

Overexpression of various PKC isoforms has been found in numerous cancers and is associated with negative disease outcomes. Here we investigated whether DENSPM treatment affects protein expression of several PKC isoforms. As shown in Figure 7, there was greater basal PKC alpha protein expression in *SDHB* *KD* cells compared to hPheo1 WT cells. Additionally, the protein expression of PKC alpha and PKC delta was reduced in hPheo1 WT cells and *SDHB KD* cells treated with DENSPM compared to vehicle treatment but not statistically different between SDHB VEH and DENSPM treatment groups (Figure 7). Protein expression between other PKC isozymes including PKC epsilon and PKC zeta was comparable between the hPheo1 cells and *SDHB KD* cells treated with DENSPM or VEH (Figures S2 and S3, respectively).

## 4 DISCUSSION

Bioactive phosphatidylethanolamines (PEs), including lyso‐PEs (LPEs), plasmenyl phosphatidylethanolamine (PE‐P), and plasmanyl phosphatidylethanolamine (PE‐O) play important roles in cancer biology. Some studies have reported these lipids can serve as helpful biomarkers. For example, one study showed high‐grade bladder cancer has high levels of plasmenyl‐phosphatidylethanolamines (Vantaku et al., 2019). Another investigation reported PEP(34:2) as a molecular marker that was detected in colorectal adenocarcinoma, but not healthy liver parenchyma (Gerbig et al., 2012). Although bioactive lipids have been studied in various types of cancer cells, the levels of various bioactive lipids have not been investigated in human cells derived from PCC. In our study, we show DENSPM, compared to vehicle treatment, reduces the levels of several plasmenyl‐phosphatidylethanolamines in hPheo1 WT and *SDHB KD* cells. The differences in bioactive lipids that we observed between the hPheo1 WT cells and *SDHB KD* cells could be due to the differences in driver mutations. hPheo1 WT cells have an NF‐1 driver mutation that causes pheochromocytoma, representing Cluster 2 mutations (Vallera et al., 2022). On the other hand, the hPheo1 *SDHB KD* cells likely represent a Cluster 1 mutation as demonstrated by Armstrong et al. (Armstrong et al., 2022).

First, we investigated whether the concentration of membrane PEs in hPheo1 WT and *SDHB KD* cells are changed by DENSPM treatment compared to vehicle treatment. Interestingly, the concentrations of multiple PE‐Ps were decreased in both the hPheo1 WT and *SDHB KD* cells with DENSPM treatment compared to vehicle treatment. Additionally, there was a greater reduction in the concentration of most PE‐Ps in the *SDHB KD* cells when compared to the hPheo1 WT cells. Both hPheo1 WT and *SDHB KD* cells were treated with the same 10 μM dose of DENSPM (equivalent to the IC50) for our lipidomic studies.

To investigate whether this IC50 dose of DENSPM was mediating apoptosis in hPheo1 WT and *SDHB KD* cells, we measured the cleaved active form of caspase‐3. Accordingly, caspase‐3 is known to have a distinct role during intrinsic apoptosis (Brentnall et al., 2013). The Western blots showed a decrease in pro‐caspase 3 and an increase in cleaved caspase 3 in hPheo1 WT and *SDHB KD* cells treated with DENSPM suggesting the drug induces apoptosis in these cells (Figure 6). The data suggest DENSPM treated *SDHB KD* cells show a higher trend in cleaved caspase 3 compared to hPheo1 WT cells.

The overexpression of various PKC isoforms has been previously reported in numerous cancers including prostate, breast, colon, stomach, thyroid and lung (Toton et al., 2011). Thus, PKC upregulation can be considered an important marker of tumorigenesis with negative outcomes. Here we show hPheo1 WT and *SDHB* *KD* cells endogenously express high amounts of PKC alpha and PKC delta isoforms. However, *SDHB* *KD* cells express significantly higher basal levels of PKC alpha compared to hPheo1 WT cells. In addition, DENSPM significantly reduced basal levels of PKC alpha and PKC delta isoforms in both hPheo1 WT and *SDHB* *KD* cells when compared to vehicle treatment (Figure 7). Basal protein expression levels between PKC zeta and PKC epsilon were comparable between the two groups (Figure S1). Presumably, PKC could have a myriad of effects in these cells including the regulation of ion channels and alteration of the actin cytoskeleton.

**FIGURE 8:**
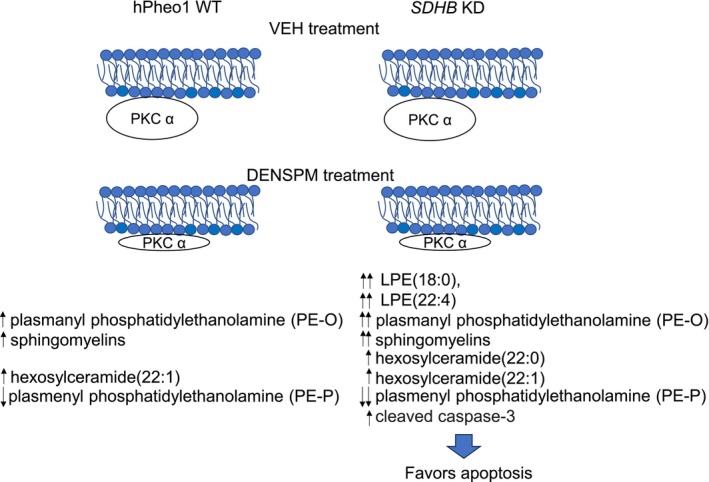
Schematic showing the effects of DENSPM treatment in hPheo1 WT and *SDHB* *KD* cells. DENSPM treatment reduced protein kinase C alpha expression and resulted in an increase in caspase‐3 dependent apoptosis in hPheo1 and *SDHB* *KD* cells. The size of the oval represents the amount of PKC alpha expression. DENSPM treatment caused an increase in the concentration of multiple forms of plasmanyl phosphatidylethanolamines (PE‐O), sphingomyelins, and hexosylceramides, but a decrease in multiple forms of plasmenyl phosphatidylethanolamines (PE‐P).

This study offers several advantages. First, it identifies several lipids that are altered by DENSPM in cells derived from human PCC (Figure 8). Second, it investigates a potential mechanism for DENSPM in hPheo1 WT and *SDHB* *KD* cells. Despite these advantages, there are some inherent limitations. First, we did not perform lipidomics using PCPG tissues with and without an *SDHB* mutation and normal tissue controls. Our goal in this study was to specifically investigate the effect of DENSPM treatment on lipids in a targeted fashion from hPheo1 WT and *SDHB KD* cells and compare the amounts of lipids from the vehicle treated group to the DENSPM treated group. Also, another limitation is that our study does not directly investigate DENSPM effects in a PCPG animal model. To address these in vivo limitations, we plan to perform a follow‐up study using an *SDHB*‐deficient mouse model and evaluate the effects of DENSPM on lipid pathways.

Taken together, the findings from this study suggest DENSPM may potentially be more effective in treating SDHB‐mutant tumors. Our lipidomic data showed a greater difference in the concentrations of multiple bioactive lipids including PE(O‐18:0/20:4), PE(O‐18:0/22:6), SM(24:0), SM(26:0), SM(18:0), HCER(22:0), HCER(22:1) in *SDHB KD* cells treated with DENSPM compared to vehicle than in hPheo1 WT cells treated with DENSPM compared to vehicle. Clinical trials are needed to determine whether DENSPM treatment is efficacious in PCPG patients with an *SDHB* mutation. Additionally, it would be interesting to investigate whether changes in bioactive phosphatidylethanolamines PEs and expression of PKC isoforms occur in human PCPG tissues with and without an *SDHB* mutation. It will be informative to see if patients treated with DENSPM have tissue or serum lipidomics profiles that correlate with data presented here.

## AUTHOR CONTRIBUTIONS

**Abdel A. Alli:** Conceptualization; formal analysis; funding acquisition; investigation; project administration; supervision. **Niharika Bala:** Data curation; formal analysis. **Yiling Xu:** Data curation; formal analysis. **Nancy D. Denslow:** Funding acquisition; methodology; resources. **Hans K. Ghayee:** Conceptualization; funding acquisition.

## CONFLICT OF INTEREST STATEMENT

HKG has received royalties from the University of Texas Southwestern Medical Center at Dallas. HKG has been a consultant for Corcept Therapeutics.

## ETHICS STATEMENT

The authors have disclosed all conflicts of interest and are committed to transparency in data management. The lipidomic dataset is contained within this manuscript.

## Supporting information


Figure S1.

Figure S2.

Figure S3.

Figure S4.

Figure S5.

Table S1.

## Data Availability

Original data generated and analyzed during this study are included in this published article or in the data repositories listed in References.
