# Supplementary figures and images for: Targeted lipidomics between hPheo1 and SDHB KD cells reveal changes in bioactive lipids and PKC with polyamine pathway inhibition
Source: Physiol Rep. 2026 Jun 5;14(11):e70894. doi: 10.14814/phy2.70894 (PMC13241704; doi:10.14814/phy2.70894)

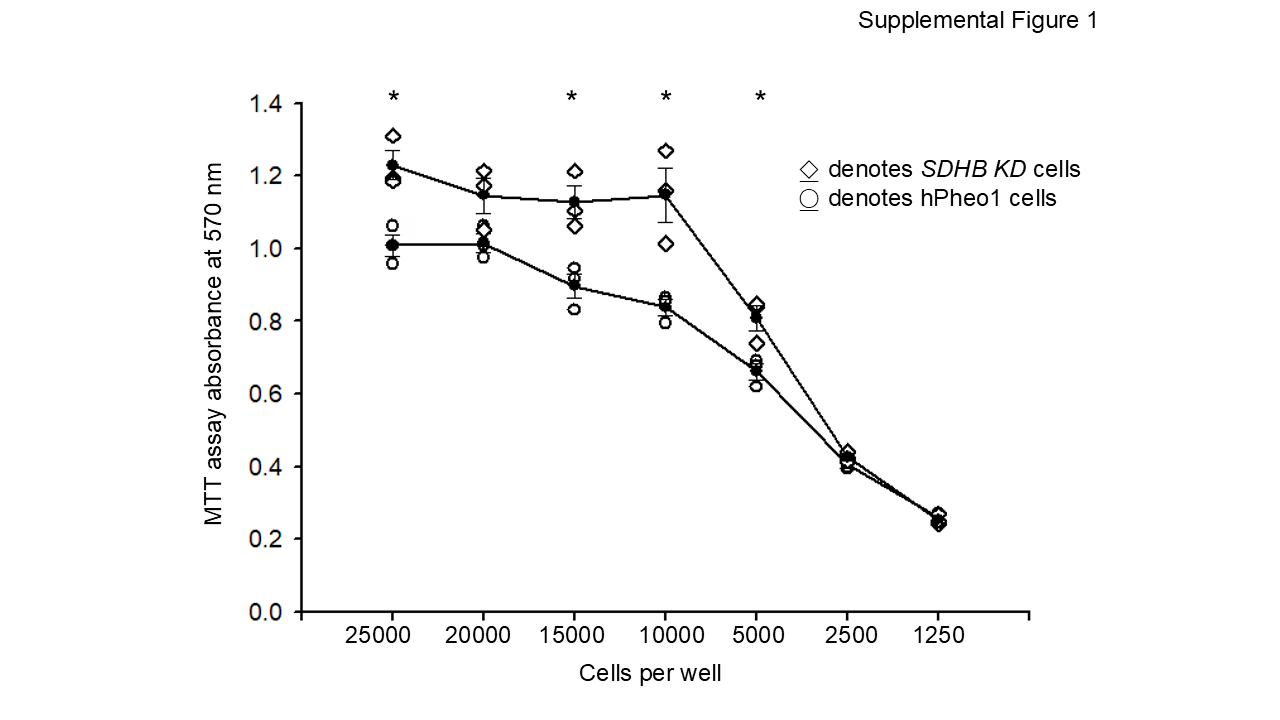

Supplement: Supplementary file 1 — Figure S1. [file PHY2-14-e70894-s006.tif]

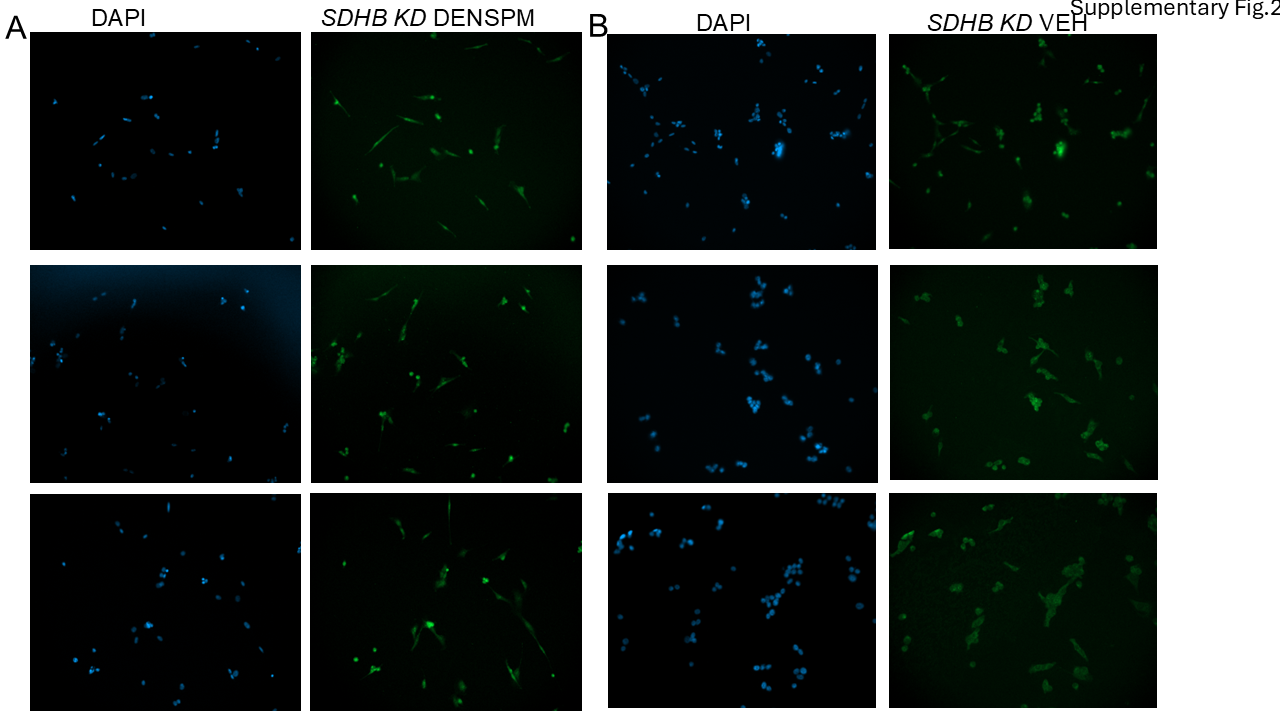

Supplement: Supplementary file 2 — Figure S2. [file PHY2-14-e70894-s002.tif]

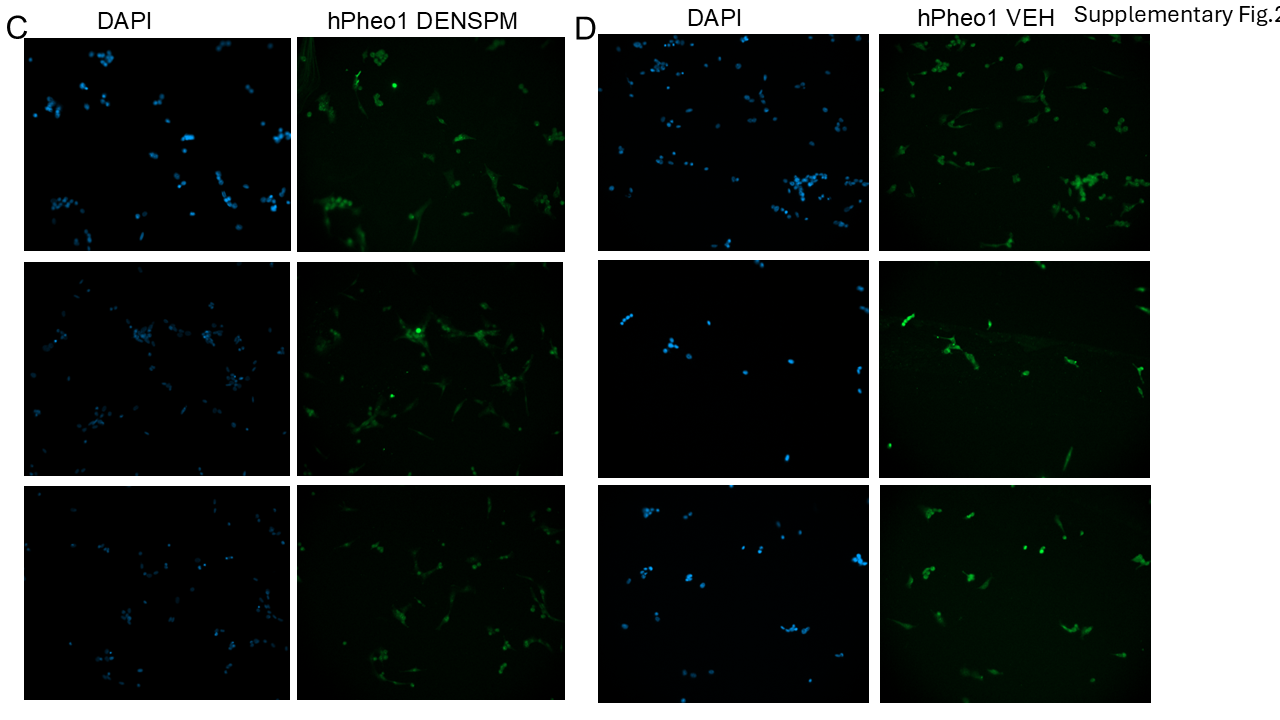

Supplement: Supplementary file 3 — Figure S3. [file PHY2-14-e70894-s004.tif]

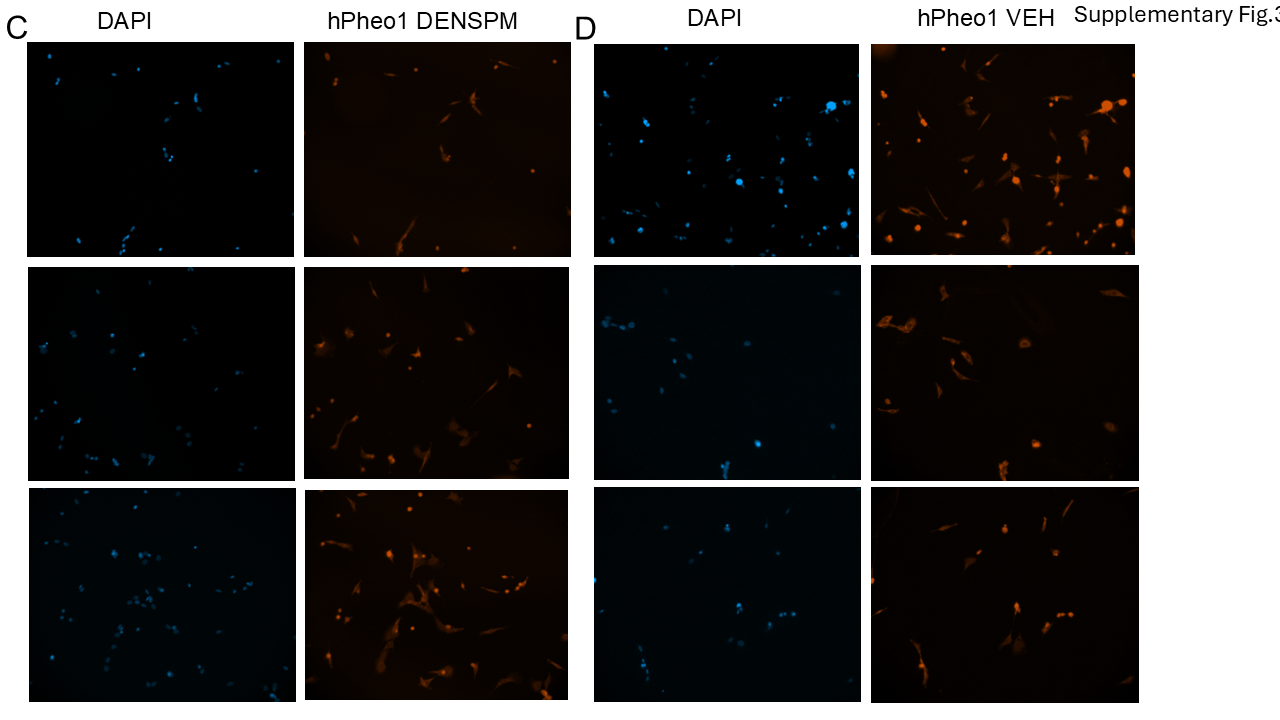

Supplement: Supplementary file 4 — Figure S4. [file PHY2-14-e70894-s003.tif]

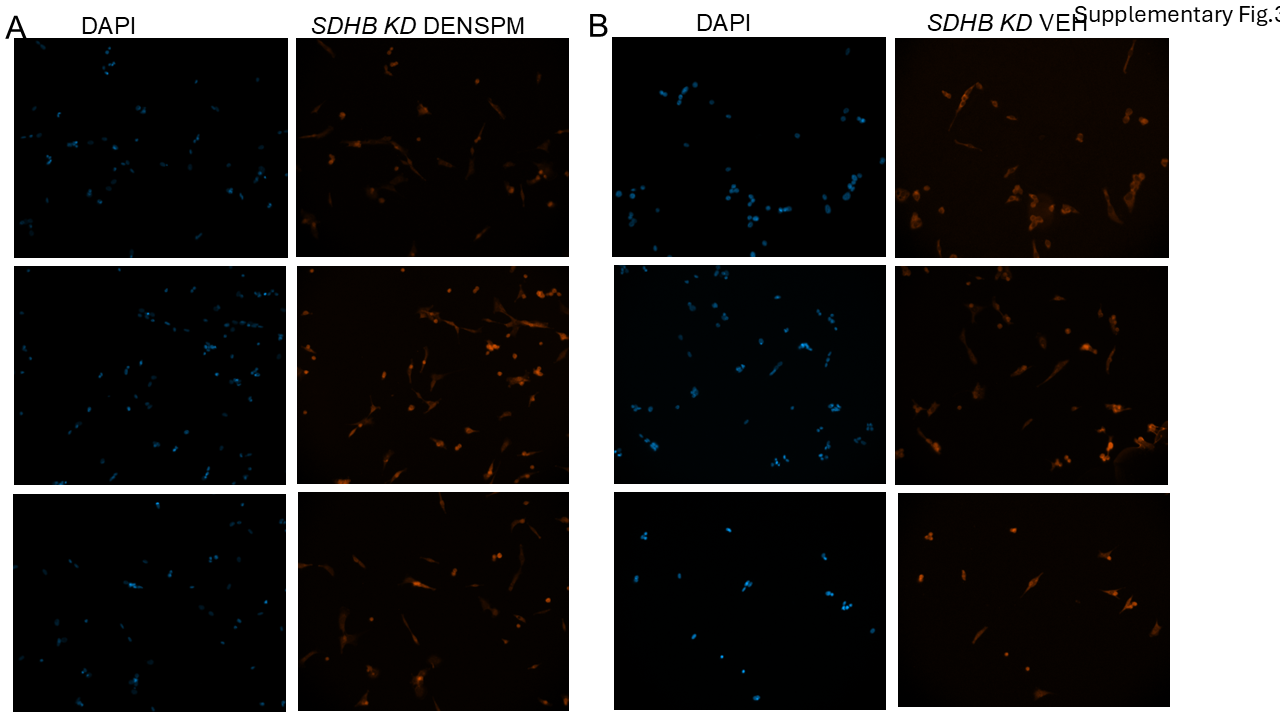

Supplement: Supplementary file 5 — Figure S5. [file PHY2-14-e70894-s005.tif]
