# Supplementary material for: Targeted lipidomics between hPheo1 and SDHB KD cells reveal changes in bioactive lipids and PKC with polyamine pathway inhibition
Source: Physiol Rep. 2026 Jun 5;14(11):e70894. doi: 10.14814/phy2.70894 (PMC13241704; doi:10.14814/phy2.70894)
Supplement: Supplementary file 6 — Table S1. [file PHY2-14-e70894-s001.docx]

Supplementary Table 1.

| **Fig 2 p-value table** |  |
| --- | --- |
| **Panel A** | **P-value** |
| sdhb veh vs. sdhb denspm | <0.001 |
| hpheo1 veh vs. sdhb denspm | <0.001 |
| sdhb veh vs. hpheo1 denspm | 0.002 |
| hpheo1 denspm vs. sdhb denspm | 0.014 |
| hpheo1 veh vs. hpheo1 denspm | 0.013 |
| sdhb veh vs. hpheo1 veh | 0.102 |
| **Panel B** | **P-value** |
| hpheo1 veh vs. sdhb denspm | <0.001 |
| sdhb veh vs. sdhb denspm | <0.001 |
| hpheo1 denspm vs. sdhb denspm | 0.009 |
| hpheo1 veh vs. hpheo1 denspm | 0.009 |
| sdhb veh vs. hpheo1 denspm | 0.01 |
| hpheo1 veh vs. sdhb veh | 0.755 |
| **Panel C** | **P-value** |
| hpheo1 veh vs. sdhb denspm | <0.001 |
| sdhb veh vs. sdhb denspm | <0.001 |
| hpheo1 denspm vs. sdhb denspm | <0.001 |
| hpheo1 veh vs. hpheo1 denspm | 0.346 |
| hpheo1 veh vs. sdhb veh | 0.603 |
| sdhb veh vs. hpheo1 denspm | 0.488 |
| **Panel D** | **P-value** |
| sdhb veh vs. sdhb denspm | <0.001 |
| sdhb veh vs. hpheo1 denspm | <0.001 |
| hpheo1 veh vs. sdhb denspm | <0.001 |
| sdhb veh vs. hpheo1 veh | 0.003 |
| hpheo1 veh vs. hpheo1 denspm | 0.002 |
| hpheo1 denspm vs. sdhb denspm | 0.077 |

| **Fig 3 p-value table** |  |
| --- | --- |
| **Panel A** | **P-value** |
| sdhb denspm vs. sdhb veh | <0.001 |
| sdhb denspm vs. hpheo1 veh | <0.001 |
| hpheo1 denspm vs. sdhb veh | <0.001 |
| hpheo1 denspm vs. hpheo1 veh | 0.002 |
| sdhb denspm vs. hpheo1 denspm | 0.029 |
| hpheo1 veh vs. sdhb veh | 0.212 |
| **Panel B** | **P-value** |
| sdhb denspm vs. sdhb veh | <0.001 |
| sdhb denspm vs. hpheo1 veh | <0.001 |
| hpheo1 denspm vs. sdhb veh | 0.001 |
| hpheo1 denspm vs. hpheo1 veh | 0.001 |
| sdhb denspm vs. hpheo1 denspm | 0.485 |
| hpheo1 veh vs. sdhb veh | 0.758 |

| **Fig 4 p-value table** |  |
| --- | --- |
| **Panel A** | **P-value** |
| sdhb denspm vs. sdhb veh | <0.001 |
| sdhb denspm vs. hpheo1 veh | <0.001 |
| hpheo1 denspm vs. sdhb veh | <0.001 |
| hpheo1 denspm vs. hpheo1 veh | 0.003 |
| sdhb denspm vs. hpheo1 denspm | 0.011 |
| hpheo1 veh vs. sdhb veh | 0.271 |
| **Panel B** | **P-value** |
| sdhb denspm vs. hpheo1 veh | <0.001 |
| sdhb denspm vs. sdhb veh | <0.001 |
| sdhb denspm vs. hpheo1 denspm | <0.001 |
| hpheo1 denspm vs. hpheo1 veh | 0.052 |
| hpheo1 denspm vs. sdhb veh | 0.054 |
| sdhb veh vs. hpheo1 veh | 0.782 |
| **Panel C** | **P-value** |
| sdhb denspm vs. sdhb veh | <0.001 |
| sdhb denspm vs. hpheo1 veh | <0.001 |
| hpheo1 denspm vs. sdhb veh | 0.003 |
| hpheo1 denspm vs. hpheo1 veh | 0.013 |
| sdhb denspm vs. hpheo1 denspm | 0.23 |
| hpheo1 veh vs. sdhb veh | 0.0237 |

| **Fig 5 p-value table** |  |
| --- | --- |
| **Panel A** | **P-value** |
| sdhb denspm vs. sdhb veh | 0.012 |
| sdhb denspm vs. hpheo1 veh | 0.174 |
| hpheo1 denspm vs. sdhb denspm | 0.738 |
| hpheo1 denspm vs. sdhb veh | 0.174 |
| hpheo1 denspm vs. hpheo1 veh | 0.738 |
| hpheo1 veh vs. sdhb veh | 0.738 |
| **Panel B** | **P-value** |
| hpheo1 denspm vs. sdhb veh | <0.001 |
| hpheo1 denspm vs. hpheo1 veh | <0.001 |
| sdhb denspm vs. sdhb veh | <0.001 |
| sdhb denspm vs. hpheo1 veh | 0.006 |
| hpheo1 denspm vs. sdhd denspm | 0.034 |
| hpheo1 veh vs. sdhb veh | 0.023 |

| **Fig 6 p-value table** |  |
| --- | --- |
| **Panel A** | **P-value** |
| hpheo1 denspm vs. hpheo1 veh | 0.0498 |
| sdhb denspm vs sdhb veh | 0.0479 |
|  |  |
| **Panel B** | **P-value** |
| hpheo1 denspm vs. hpheo1 veh | 0.0444 |
| sdhb denspm vs sdhb veh | 0.0096 |

| **Fig 7 p-value table** |  |
| --- | --- |
| **Panel A** | **P-value** |
| hpheo1 denspm vs. hpheo1 veh | 0.0431 |
| sdhb denspm vs sdhb veh | 0.0319 |
| sdhb veh vs. hpheo1 veh | 0.039 |
|  |  |
| **Panel B** | **P-value** |
| hpheo1 denspm vs. hpheo1 veh | 0.0411 |
| sdhb denspm vs sdhb veh | 0.0927 |
| Hpheo1 veh vs. sdhb veh | 0.0629 |
